# Initial experience with a synthetic sealant PleuraSeal™ after pulmonary resections: a prospective study with retrospective case matched controls

**DOI:** 10.1186/1749-8090-5-50

**Published:** 2010-06-16

**Authors:** Sebastian Dango, Rong Lin, Ellen Hennings, Bernward Passlick

**Affiliations:** 1 Department of Thoracic Surgery, University Medical Centre Freiburg, 79106 Freiburg, Germany; 2 Biostatistical Consulting Inc., 10 Mall Road, Suite 200, Burlington, MA 01803, USA

## Abstract

The objective of this study was to evaluate postoperative outcome and efficacy of a hydrogel tissue sealant for prevention of alveolar leakage after open lung resections.

20 consecutive patients were enrolled in the PleuraSeal™ sealant group (PSG) and case matched with 20 retrospective controls (CG) with standard treatment. Assessment of postoperative air leakage was performed until chest tube removal. Patients were followed until 30 days after discharge.

At end of surgery, 100% in the PSG and 0% in the CG were air leak free (p < 0.001). Duration of postoperative chest tube suction was shorter in PSG (p < 0.001), and air leak chest tube was removed earlier (p = 0.03). Limitation for chest tube removal due to a pulmonary leak was 35% in CG and 5% in PSG (p = 0.04). Patients remaining air leak free thru discharge was 95% and 15% for PSG and CG (p < 0.001).

The study demonstrated a superior efficacy of PleuraSeal™ sealant compared with standard surgical treatment for sustained sealing of postoperative air leakage and causes shorter air leak chest tube duration.

## Introduction

Postoperative air leakage remains the most common pulmonary complication in patients undergoing pulmonary resection with a reported occurrence of 18-58% of the cases [1,2]. Persistent postoperative air leakage (>7 days) has been reported in up to 25% of patients undergoing pulmonary resection [3]. Described risk factors for pulmonary leakage are incomplete fissures [4], underlying lung diseases such as emphysema, fibrosis, tuberculosis or malignancies [5], presence of a lymphangioleiomyomatosis [5], intrathoracic adhesions [6], older patients (>75 years) [7], and lower diffusion capacity [8]. Deleterious effects of prolonged air leaks are longer chest tube duration often associated with prolonged pain [6,9], prolonged hospitalization [6,8,10] and greater health care costs [9,10], increased risk of pneumonia and empyema [8,9], decreased postoperative mobility [11], and can necessitate pleurodesis or re-operation [11]. In addition, the incidence of postoperative empyema increases dramatically when air leak is present. As shown by Brunelli et al [12], postoperative empyema was found in 1% and 10% of patients with pulmonary leaks less than and greater than 7 days, respectively.

Surgical suturing and stapling are the standard methods for the prevention and treatment of air leakage and can be challenging especially in patients with fragile lung tissue or emphysema as seen in smokers. Over the last decade, various surgical sealants have been introduced for further prevention and reduction of air leaks. These include liquid autologous fibrin-based sealants [13,14], collagen fleece-bound sealants [15,16], and polyethylene glycol (PEG) hydrogel sealants such as PleuraSeal™ [17,18]. PleuraSeal™ lung sealant is a new, easy to prepare, 100% synthetic, flexible resorbable blue gel that expands with lung inflation and has a sealing strength five-times greater than normal liquid fibrin glue [19]. PleuraSeal™ sealant begins as two liquids that crosslink when mixed; rapidly changing within seconds into a solid hydrogel that mechanically bonds to the underlying tissue. This hydrogel remains in place while natural healing occurs underneath the gel, hydrolyzing like absorbable sutures within 4-8 weeks. The objectives of this study were to evaluate the postoperative outcome, safety, and efficacy of this new hydrogel tissue sealant for the sealing of alveolar leakage after lung resections.

## Patients and Methods

### Setting and samples

To investigate the efficacy and postoperative outcome, PleuraSeal™ lung sealant system was prospectively evaluated as an adjunct to standard closure techniques for control of visceral pleural air leaks in patients scheduled for pulmonary resection. These data were compared with an individually case matched retrospective control cohort. Sample size calculation was based on a superiority test for two proportions using Fisher's Exact Test, estimated proportion was 72% for PSG and 15% for CG, respectively. Therefore, for an estimated power of 90% a sample size of 18 patients for each group was necessary to show a difference in the two-sided test for the primary endpoints to reach statistical significance. Data were collected at the University Hospital Freiburg, Department of Thoracic Surgery, from patient admission to discharge. Twenty consecutive patients with anatomical resection were included. Inclusion criteria for both groups were patient age 18 years or older, and a scheduled or completed (in retrospective control group) lobectomy or segmental wedge resection via an open thoracotomy approach and presence of an initial intraoperative air leak after resection. Preoperatively, groups were well balanced regarding concomitant pulmonary disease, mean FEV1, lung tissue quality, presence of diabetes and smoking habit. The study was approved by the local ethical committee (340/08, 12/02/08) and written informed consent of all patients was obtained. Only patients with intraoperative air leaks grade 1-2 based on the Macchiarini scale [20] after standard surgical suture or stapling were enrolled. All subjects had two chest tubes during the first 48 hours after thoracotomy. Routinely two silicone chest tubes (ventral 21 Ch and dorsal 24 Ch, silicone chest tubes, Redax Company, Mirandola, Italy) were placed after thoracotomy and connected to a water sealed drainage system. Suction of -20 cm H_2_O was taken off once no air leaks were detected after 12 hours of observation time. The ventral chest tube (air leak chest tube) was taken out when there was no evidence of an air leak within the last 24 hours, and the dorsal tube was taken out when less than 200 ml drainage was recorded within last 24 hours. Assessment of postoperative air leakage was performed twice daily until chest tube removal and discharge. Patients were discharged and re-evaluated within 30 days after leaving the hospital, with two fixed scheduled visits to an ambulatory outpatient clinic. Primary criteria for discharge were no significant pain (VAS ≤ 2) under present pain medication and absence of chest tube.

### Application and technique of PleuraSeal™ Lung Sealant System

Prior to the application of PleuraSeal™ lung sealant an intraoperative air leak test during lung inflation was carried out to evaluate the location and grade of air leaks as described above. In the event of a grade 3 air leak additional suture or stapling was performed to downgrade the pulmonary leak, so that PleuraSeal sealant was applied only in patients with grade 1 or 2 leaks. PleuraSeal™ polymer kit with a total volume of 5 ml was applied using a MicroMyst™ Applicator with a continuous flow (flow regulator) under aseptic conditions to seal intraoperative air leaks as well as prophylacticly on staple lines and other manipulated lung tissue. The lung surface was as dry as possible and the lung was inflated between 50-75% of its maximal volume. The product was uniformly distributed in a layer of 1-2 mm thickness, and on areas of extending pulmonary lesions a thicker layer was achieved of up to 3 mm. Excessive material was mechanically removed using suction or blunt dissection. Contraindications to apply PleuraSeal™ lung sealant include uncontrolled transected bronchioles >1 mm, and intrathorax infections. Additionally, the sealant was not applied to bronchial stumps or bronchial anastomoses. Following sealant application leak sites underwent another water submersion test under pressure of 25 mmHg. Sealant application could be repeated if air leakage control was not halted after the first application.

### Clinicopathological Parameters

Concerning overall clinical characteristics, a total of 20 consecutive patients in the PleuraSeal™ sealant study group were matched with 20 retrospective patients that underwent pulmonary resection via an anterolateral thoracotomy as a control group. Baseline characteristics and intraoperative findings are shown in Table 1 and 2, respectively without any statistical difference between the two groups (Table 1. and 2.).

**Table 1:** Baseline characteristics and pre-operative variables

**Variable **^**a**^	Overall	PleuraSeal™ Group	Control Group	***p*-value**^**b**^
	**40**	**20**	**20**	

**Age (yrs.), mean (SD)**		69 (45-82)	66 (47-81)	*0.34*

**Gender**				
Male	31 (77%)	14 (70%)	17 (85%)	
Female	9 (22%)	6 (30%)	3 (15%)	*0.45*

**Chronic lung disease**	**22**	**11**	**11**	
COPD	19 (47%)	9 (45%)	10 (50%)	
Lung emphysema	2 (5%)	2 (10%)	0 (0%)	
Asthma	1 (2%)	0 (0%)	1 (5%)	*0.58*

**Pulmonary function **^**c**^				
FEV1 actual (Ø %), mean (SD)	85.5 (17.9)	87 (20.2)	84 (15.6)	*0.51*
FEV1 predicted (Ø %), mean (SD)	65.5 (16.2)	69 (17.6)	62 (14.8)	*0.18*
DLCO actual (Ø %), mean (SD)	70.5 (14.5)	70 (15.7)	71 (13.3)	*0.84*
DLCO predicted (Ø %), mean (SD)	55 (14.6)	56 (16.3)	54 (12.9)	*0.55*

**Neoadjuvant therapy**	**1**	**0**	**1**	
Radiation	0 (0%)	-	-	*NA*
Chemotherapy	1 (2%)	0 (0%)	1 (5%)	*1.00*

**Concomitant disease**	**23**	**14**	**9**	
Diabetes	7 (17%)	4 (20%)	3 (15%)	*1.00*
Others^d^	16 (40%)	10 (50%)	6 (30%)	*0.23*

**Smoking behaviour**				
Current Smoker	15 (37%)	8 (40%)	7 (35%)	
Ex smoker (≥12 month)	13 (32%)	6 (30%)	7 (35%)	
Never smoked	12 (30%)	6 (30%)	6 (30%)	*0.14*

^**a **^Numbers in percentage were rounded up were indicated.

^**b **^Continuous variables were compared between groups by Students' t-test. Categorical variables were compared using two-sided Fisher's Exact test..

^**c **^Preoperative pulmonary function test and postoperative variables based on amount of lung resection; FEV1: Forced expiratory volume (1 sec); DLCO: CO volume-adjusted diffusion capacity). Data shown in % of total function and as mean with SD where indicated; SD standard deviation.

^**d **^Other entities: hypertension, cardiac insufficiency, arrhythmia absoluta

**Table 2:** Surgical variables and intra-operative findings

**Variable **^**a**^	Overall	PleuraSeal™ Group	Control Group	***p*-value **^**b**^
	**40**	**20**	**20**	

**Surgical diagnosis**				

Lung cancer	38 (95%)	18 (90%)	20 (100%)	
Lung metastasis	2 (5%)	2 (10%)	0 (0%)	*0.48*

**Operation time (hh:mm)**		02:35	03:07	*0.07*

**Lung tissue quality**		**20**	**20**	
Normal	21 (52%)	10 (50%)	11 (55%)	
Fragile	19 (47.5%)	10 (50%)	9 (45%)	*1.00*

**Procedure**				
**Right lobectomy**	**19 (47.5%)**	**7 (35%)**	**12 (60%)**	
Upper	13 (32%)	6 (30%)	7 (35%)	
Middle	0 (0%)	0 (0%)	0 (0%)	
Lower	6 (15%)	1 (5%)	5 (25%)	
**Left lobectomy**	**18 (45%)**	**11 (55%)**	**7 (35%)**	
Upper	10 (25%)	7 (35%)	3 (15%)	
Lower	8 (20%)	4 (20%)	4 (20%)	
**Segmentectomy**	**2 (5%)**	**1 (5%)**	**1 (5%)**	
**Bilobectomy**	**1 (2.5%)**	**1 (5%)**	**0 (0%)**	*0.26*

**Intraoperative air leaks characteristics**				
Intraoperative leak free (in %)		100%	0%	***<0.001***
Postoperative leak free (in %)^c^		95%	15%	***<0.001***
Initial # of intraoperative air leaks/patient (Ø)^d^	1.6	1.6	1.6	*1.00*
Initial grade of air leaks/patient	1.35	1.3	1.4	0.77

**Additional procedure for air leak control**	**8**	**2**	**6**	
Suture	8 (20%)	2 (10%)	6 (30%)	*0.23*
Staple	0 (0%)	-	-	*NA*

^**a **^Numbers in percentage were rounded up were indicated.

^**b **^Continuous variables were compared between groups by Students' t-test. Categorical variables were compared using two-sided Fisher's Exact test.

^**c **^Leak free at the end of surgery

^**d **^Based on Macchiarini scale

### Efficacy and safety endpoints

Three primary endpoints were defined by protocol: reduction of intraoperative air leakage, the presence and duration of postoperative air leak, and postoperative morbidity. Secondary parameters were duration of chest tube placement as well as time to discharge. Adverse events (AE) for the prospectively collected study cohort from the screening period prior to admission to our surgical ward until follow-up of 30-days after discharge were documented and reported if eligible. Complications were defined as occurrence of empyemas, incomplete lung inflation, pneumothorax, presence of bronchogenic fistula, chylothorax, cardiovascular complications, any other organ specific failure, severe postoperative pain, acute esophagitis, and the need for additional procedures (re-operation, pleurodesis, and replacement of chest tube).

### Statistical analyses

All statistical analyses were performed using SAS^® ^Version 9.1. All statistical tests were two-sided at the 5% significance level. Continuous variables were summarized using mean and standard deviation, and the two sample t-test was used to test the difference between treatment groups. Categorical variables were summarized by frequencies and percentages, and the proportions were calculated and compared between treatment groups using Fisher's Exact test.

## Results

### Trial treatment and control group

In the PleuraSeal™ sealant group two patients failed to have any intraoperative air leaks and were excluded from the study after screening. Conversely 91% of prospective lobectomy patients (20/22) demonstrated at least one intraoperative air leak. The twenty study patients received primary PleuraSeal™ sealant treatment after a water submersion test identified air leak location. The majority of air leaks occurred in the interfissure area (52%), with 23% at the staple lines, 6% at the suture lines, 6% due to adhesiolysis, and the remaining 13% due to other tissue manipulation (Table 3). For each patient the number of air leaks were counted and graded. Prior to the application of PleuraSeal™ sealant, the grade of air leak was 1.2 +/- 0.4 (mean +/- SD) in the PSG which was comparable to 1.1 +/- 0.2 (mean +/- SD) in CG. (p = 0.24). Also prior to the application of PleuraSeal™ sealant, the number of intraoperative air leaks per patient was 1.6 +/- 0.6 (mean +/- SD) in the PSG which was comparable to 1.4 +/- 0.5 (mean +/- SD) in CG (p = 0.39). In both groups additional suturing was carried out if necessary to downgrade air leaks prior to PleuraSeal™ sealant application in the PSG and closure in the CG. This was performed in 2 cases (10%) in the PSG and 6 cases (30%) in the CG without any significant difference between the two groups (p = 0.23). Neither in the PSG nor CG did we perform any additional stapling. On average 2.8 ml of PleuraSeal™ sealant was applied in less than one minute to the air leak sites and prophylacticly to all manipulated tissue. The MicroMyst™ applicator enabled multiple starts and stops during application without clogging. Only one patient needed a second application of PleuraSeal™ sealant. The mean operating time in the PSG was 2.6 hours which was comparable to the 3.1 hours in the CG (p = 0.08).

**Table 3:** Application of PleuraSeal™ sealant

**Variable **^**a**^	PleuraSeal™ Group	Control Group	***p*-value**^**b**^
	**20**	**20**	

**Adjusted air leaks**			

Total # of air leaks	31	28	*NA*
Initial # of air leaks/patient	1.6 (0.7)	1.6 (0.5)	*1.00*

# of air leaks/patient after additional treatment	1.6 (0.6)	1.4 (0.5)	*0.39*

Initial grade of air leaks/patient	1.3 (0.6)	1.4 (0.5)	*0.77*

Grade of air leaks/patient after additional treatment	1.2 (0.4)	1.1 (0.2)	*0.24*

**PleuraSeal™ application**	**20**	-	*NA*
Volume of sealant (ml)^c^	2.8 (1.0)	-	-
Duration (s)^c^	43.0 (17.7)	-	-
Second application (n)	1 (5%)	-	-
Micromyst applicator	20 (100%)	-	-

**Air leak locations**			*NA*
Staple lines	7 (23%)	-	-
Suture lines	2 (6%)	-	-
Interfissure area	16 (52%)	-	-
Adhesiolysis	2 (6%)	-	-
Other manipulation	4 (13%)	-	-

^**a **^Numbers in percentage were rounded up were indicated.

^**b **^Continuous variables were compared between groups by Students' T-test. Categoric variables were assessed by two-tailed Pearson's Chi square test.

^**c **^Data shown as mean with standard deviation (SD), # number of air leaks.

### Postoperative outcome

Overall morbidity was 52.5% and we could not find a statistical difference between the two study groups (*p *= 0.60) (Table 4). In total, 4 (10%) patients in the combined PSG and CG had severe pain postoperatively defined by a visual analogue scale (VAS) score ≥4 under present pain medication, in 5 (12.5%) patients cardiac complications were observed, and only one patient needed postoperative mechanical ventilation after onset of an adult respiratory distress syndrome. We did not experience any pleural empyema, lung emboli, incomplete lung inflation, re-thoracotomies, or re-placement of a chest tube in either of the groups. No patient died during the study period. All patients were discharged from hospital and completed the follow up period without any late complications. Hospital length of stay was 9.9 days in the PSG vs. 11.7 days in the CG, however, it did not reach statistical significance (p = 0.178). An interesting observation is that the shorter hospital length of stay of 1.8 days in the PSG corresponds exactly to the 1.8 days earlier removal of the air leak chest tube in the PSG.

**Table 4:** Post-operative complications and outcome

**Variable **^**a**^	Overall	PleuraSeal™ Group	Control Group	***p*-value**^**b**^
	**40**	**20**	**20**	

**Duration of chest tube (d) **^**c**^, mean (SD)				

Ventral chest tube removal (air leak)		2.1 (1.2)	3.9 (3.3)	***0.03***
Dorsal chest tube removal (drainage)		5.1 (4.0)	5.2 (3.4)	*0.94*

**Chest tube on suction (h) **^**c **^mean (SD)		22.9 (1.8)	49.7 (28.2)	***<0.001***

**Limiting factor for chest tube removal**				
Air leakage	8 (20%)	1 (5%)	7 (35%)	***0.04***
Drainage	32 (80%)	19 (95%)	13 (65%)	*0.94*

**Total # of postoperative complications **^**d**^	**21 (52.5%)**	**10 (50%)**	**11 (55%)**	**1.000**
Bronchogenic fistula	1 (2.5%)	0 (0%)	1 (5%)	
Chylothorax	3 (7.5%)	2 (10%)	1 (5%)	
Pneumonia	6 (15%)	4 (20%)	2 (10%)	
Mechanical Ventilation/ARDS	1 (2.5%)	0 (0%)	1 (5%)	
Tachyarrhythmia absoluta	5 (12.5%)	3 (15%)	2 (10%)	
Postoperative severe pain	4 (10%)	1 (5%)	3 (15%)	
Others^e^	1 (2.5%)	0 (0%)	1 (5%)	

**Length of stay(days) **^c ^				
Potential hospitalization		8.2 (4.6)	10.3 (5.5)	0.205
Actual hospitalization		9.9 (3.7)	11.7 (4.4)	0.178

^**a **^Numbers in percentage were rounded up were indicated.

^**b **^Continuous variables were compared between groups by Students' T-test. Categoric variables were assessed by two-tailed Pearson's Chi square test.

^**c **^Data shown as mean with standard deviation (SD)

^**d **^# total number of events

^**e **^Other complications: acute esophagitis.

### Intraoperative and postoperative air leakage and chest tube duration

**Figure 1:**
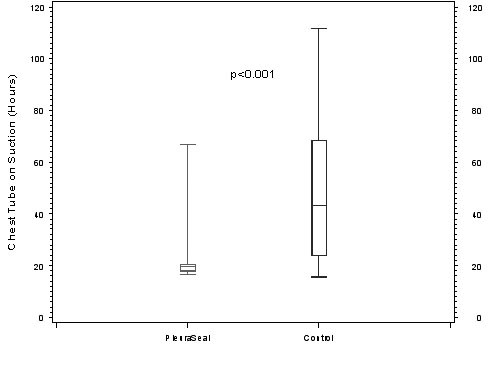
**Time of chest tube suction**.

**Figure 2:**
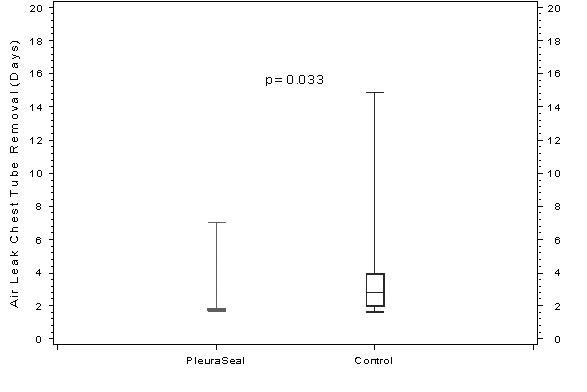
**Duration of postoperative chest tube drainage for air leakages**.

At the end of surgery, 100% of the patients treated with the synthetic PleuraSeal™ sealant and 0% of the control group were air leak free (p < 0.001). One hour after surgery thru 30 day follow-up, 95% of the patients in the PSG were still air leak free compared to 15% in the CG (p < 0.001). After admission to our post operative unit all chest tubes were treated with suction as defined by protocol. The duration of postoperative air leak chest tube suction was significantly shorter in the PSG compared to the CG (Figure 1, p < 0.001), and air leak chest tube (ventral chest tube) was removed significantly earlier at 2.1 days for PSG compared to 3.9 days for CG (Figure 2, p = 0.03). Limitation of chest tube removal due to a pulmonary leak was 5% in PSG and 35% in CG (p = 0.04) without any difference for drainage time (p = 0.94). All patients in the PSG had no evidence of pneumothorax in chest X-ray before discharge and after a 30 day follow-up period compared with 4 (20%) in the CG (p = 0.16).

## Conclusion

A simple, quick, and reproducible method to seal air leaks during thoracic procedures for lung resections could have a great impact on postoperative air leakage and clinical outcome. Ideally, such a product should bind rapidly to the tissue with strong adherence and not constrict lung volume expansion. It should be bacteriostatic and non-irritating, slowly and controllably break down in body fluids and be systemically non-toxic and inherently safe [9,21]. The trial sealant PleuraSeal™ used in the study has been shown to have very good biocompatibility and has undergone extensive pre-clinical testing both in vitro [19], and in vivo [21] Furthermore, its components have been used in several medical applications [22,23] and it has been proven to have strong adherence and flexibility in an ex-vivo porcine lung model, sealing coin size defects measuring approximately 5 mm in depth and 15 mm in diameter and with lung expansion up to 40 cm H2O. In this model, the trial sealant demonstrated 100% adherence and expansion without sealant tearing or delamination, and without restricting the normal expansion of the lung. The average burst strength pressure for the PleuraSeal™ sealant applied with the MicroMyst™ air-assisted applicator over the coin size defects was 291 cm H2O, 167 cm H2O, and 156 cm H2O at 0, 24, and 48 hours, respectively, which exceeds the maximal cough pressure at 2 days post-op of 102 cm H2O [24]. The mode of failure in all instances was cohesive rather than adhesive.

The study demonstrated a superior efficacy of the trial sealant as compared with standard surgical treatment for sustained sealing of postoperative air leakage following pulmonary resections. It enables an immediate and safe air tight seal intraoperatively with a high proportion of patients remaining air leak free, shorter air leak chest tube suction and duration, and may lead to a decrease in hospital length of stay. However, many factors can impact the length of stay for thoracic patients: postoperative air leaks, co-morbidities, the patient's social environment and healthcare reimbursement incentives. It should be noted that under the German Healthcare System there is currently no financial incentive to discharge patients from the hospital as soon as possible, as there is in the US system. One plausible explanation for the tendancy of shortened hospital length of stay in this study is that the earlier removal of the air leak chest tube and attached canister in the PSG increased patient mobility leading to a faster recovery. It is interesting that a shorter hospital length of stay of 1.8 days in the PSG corresponds exactly to the 1.8 days earlier removal of the air leak chest tube in the PSG.

However, the study has its limitations as far as the included study population is concerned. Since this is a preliminary study after introduction of this surgical device in patients undergoing lung resections only a small number of 20 patients as calculated above were included and compared to a case-match control cohort. Of note is that we could demonstrate statistical significance of p < 0.001 for our primary outcomes and showed reduced intraoperative air leakage and postoperative leak free through discharge.

The ease of use and effective sealing attributes of the PleuraSeal™ sealant enables a surgeon to change their mindset toward alveolar air leaks, and strive toward intra-operative leak free surgery. It is common practice to fill the chest cavity with warm saline to check intraoperatively for bronchopleural fistula and during this step one may also accurately identify the location of alveolar air leaks by gradually lowering the water level and identifying the source of air bubbles. The sealant may then be applied site specifically to cover all the identified air leaks and also prophylacticly to staple lines and manipulated tissue in less than one minute. Especially, since 52% of the intra-operative air leaks in the study were detected in the interfissure area which is difficult to access by standard suturing or fleece-bond sealants, PleuraSeal™ as a liquid sealant is ideal to seal air leaks in this interlobar space with its many anatomical variations. As part of this Protocol we resubmerged the lung parenchyma with warm saline and retested under pressure to confirm that all alveolar air leaks had been successfully halted with the study sealant, and in only one patient was an additional application of the trial sealant required to obtain intra-operative leak free surgery. We did not experience any pleural empyema, pneumothoraces, chest tube replacement, or re-operation in the presented study.

In summary, this liquid, hydrogel tissue sealant is safe and easy to use and has a significant impact on intra- and postoperative air leakage prevention as well as on early chest tube removal for air leaks. We also found a trend toward a reduced length of hospitalization, but this did not reach statistical significance.

## Competing interests

The authors declare that they have no competing interests.

## Authors' contributions

SD participated in the study design, collected patient samples and performed data acquisition, analysed the data, drafted the manuscripts, and is the corresponding author. RL carried out statistical analysis. EH collected patient samples and carried out data acquisition. BP reviewed the study design and manuscript draft. All authors have read and approved the final manuscript.
